# Supplementary material for: Cultivating Healthier Habits: The Impact of Workplace Teaching Kitchens on Employee Food Literacy
Source: Nutrients. 2024 Mar 16;16(6):865. doi: 10.3390/nu16060865 (PMC10975800; doi:10.3390/nu16060865)
Supplement: Supplementary file 1 [file nutrients-16-00865-s001.zip › nutrients-2847544-supplementary.pdf]

Pre and Post-class Surveys

Importance of the Learning Experience

1. Please indicate your level of agreement with the following statements.

	Strongly disagree	Disagree	Neither agree or disagree	Agree	Strongly agree
A. I want to learn about how to prepare and cook new foods and cuisines	○	○	○	○	○
B. It is important to eat food made with healthy ingredients	○	○	○	○	○
C. It is important to eat food that has been grown sustainably	○	○	○	○	○
D. It is important to eat foods that are in season	○	○	○	○	○
E. I want to know more about how foods can improve my wellbeing	○	○	○	○	○
F. It is important to eat home-cooked food	○	○	○	○	○
G. I want to know more about how my food choices can affect the environment	○	○	○	○	○

Ease and Pleasure of Cooking

2. Please indicate your level of agreement with the following statements.

	Strongly disagree	Disagree	Neither agree or disagree	Agree	Strongly agree
A. I can fit cooking into my schedule	○	○	○	○	○
B. I enjoy cooking	○	○	○	○	○
C. I find it simple to cook food	○	○	○	○	○
D. I can easily find the foods that meet my needs	○	○	○	○	○
E. I enjoy eating the food I make	○	○	○	○	○

Food Skills

3. Please tell us how you would rate your food skills today.

Use the slider to tell us how you would rate your food skills today

0 1 2 3 4 5

Plan meals ahead of time

Prepare a meal for a special occasion

Prepare meals that include vegetables, grains/starches, legumes/pulses or other protein-rich foods

Cooking techniques (sauteing, oven-roasting, steaming ...)

Knife skills (chopping, slicing, dicing ...)

Adapt recipes to make them healthier

Try a new recipe

Prepare the right amount of food

Include a variety of vegetables and fruit with meals

Note: 0 was not selectable - only the integers 1-5 were selectable.

Confidence in the Kitchen

4. HOW CONFIDENT do you feel about performing each of the following activities?

	Not confident at all	Slightly confident	Somewhat confident	Fairly confident	Completely confident
A. Eating the Recommended amount of vegetables and fruit each day	○	○	○	○	○
B. Planning a balanced meal	○	○	○	○	○
C. Preparing a tasty meal using mainly fresh and whole food ingredients	○	○	○	○	○
